# The role of temporal distance of the events on the spatiotemporal dynamics of mental time travel to one’s personal past and future

**DOI:** 10.1038/s41598-022-05902-8

**Published:** 2022-02-11

**Authors:** I. Colás-Blanco, J. Mioche, V. La Corte, P. Piolino

**Affiliations:** 1 Laboratoire Mémoire, Cerveau et Cognition (MC2Lab), UR 7536, Université de Paris, 71 Avenue Edouard Vaillant, Boulogne-Billancourt, Île de France France; 2 Institut Universitaire de France (IUF), Paris, France; 3 Institut de la Mémoire et de la Maladie d’Alzheimer (IM2A), Département de Neurologie, Hôpital Pitié-Salpêtrière, AP-HP, Paris, France

**Keywords:** Psychology, Human behaviour, Cognitive neuroscience, Neuroscience, Long-term memory

## Abstract

Mental time travel to personal past and future events shows remarkable cognitive and neural similarities. Both temporalities seem to rely on the same core network involving episodic binding and monitoring processes. However, it is still unclear in what way the temporal distance of the simulated events modulates the recruitment of this network when mental time-travelling to the past and the future. The present study explored the electrophysiological correlates of remembering and imagining personal events at two temporal distances from the present moment (near and far). Temporal distance modulated the late parietal component (LPC) and the late frontal effect (LFE), respectively involved in episodic and monitoring processes. Interestingly, temporal distance modulations differed in the past and future event simulation, suggesting greater episodic processing for near as opposed to far future situations (with no differences on near and far past), and the implementation of greater post-simulation monitoring processes for near past as compared to far past events (with high demands on both near and far future). These findings show that both past and future event simulations are affected by the temporal distance of the events, although not exactly in a mirrored way. They are discussed according to the increasing role of semantic memory in episodic mental time travel to farther temporal distances from the present.

## Introduction

Mental time travel, that is, the ability to remember our personal past and imagine our personal future, is thought to rely on the association of perceptual, affective, and spatiotemporal contextual details that enable the (p)re-experiencing of specific events in our life. It has been proposed that mental time travel to the past (episodic autobiographical memory, EAM) and to the future (episodic future thinking, EFT) rely on the same constructive simulation process^1^, as both involve the generation of complex and multimodal event representations that require event content integration with phenomenal details. In particular, the following three cognitive processes seem to be crucial to mental time travel in both temporal directions: the processing of self-reference information, visual imagery, and episodic binding. These processes engage the medial prefrontal cortex (mPFC), the precuneus, and the hippocampus, respectively, brain areas reported in EAM and EFT studies^2–4^.

Recently, a large body of evidence has pointed to the fact that EAM and EFT partly rely on the same core brain system, a network including the frontal poles, the medial temporal lobes (MTL), the posterior cingulate, precuneus and retrosplenial cortex, and lateral parietal and temporal areas^5–8^. Further evidence indicating that both processes are subserved by the same cognitive and neural systems shows that deficits in EAM correlate with impairments in EFT, as age-related memory decay similarly impacts the quality of past and future mental evocations (reducing the number of episodic details^9–11^), and hippocampal lesions affect both remembering one’s past and foreseeing one’s future^12–15^.

Despite sharing the above-mentioned neural and cognitive correlates, past events are usually reported as being richer in contextual and sensorial details than future events, whereas future events tend to present a more positive emotional valence than past events^12,16,17^. Interestingly, the temporal distance of the simulated events to the present moment can also modulate their phenomenological properties^9,17–24^. It is known, for instance, that most episodic autobiographical memories go through a process of semanticization over time^25–27^, losing the spatiotemporal context in which they were acquired. This abstraction process is usually accompanied by a neural disengagement from the hippocampus at memory retrieval^28^ and a shift to reliance on neocortical activation. An exception has been observed for highly self-relevant and/or emotional memories, which can resist this abstraction process, remaining episodic in nature and dependent on hippocampal activation^29–32^. In the light of the semantic scaffolding hypothesis of EFT, thinking directed towards the future also relies on semantic representations^22^. Furthermore, some theories such as the temporal construal^33,34^ and the TEDIFT^20^model (Temporal Distance in Future Thinking) propose that representations of distant events are more abstract or generic than representations of events closer to the present moment, regardless of the temporal direction (past or future) in which those events take place. Therefore, some authors have posited that, like past memories, the prospection of distant events is more semantic^20^ or less hippocampal dependent^35^ than the prospection of events that are closer to the present, except for highly self-relevant or emotionally positive future events. Nevertheless, the experimental results on this issue are not homogeneous yet.

In a pioneer positron emission tomography (PET) study, Okuda et al.^5^ observed similar temporal distance modulations in the majority of the frontotemporal regions evaluated when participants were freely talking about past as well as future situations happening at near as compared with far temporalities (i.e., a few days vs a few years). Since then, a considerable number of functional magnetic resonance imaging (fMRI) studies have explored the neural correlates of mental time travel to the past and the future^4,36–38^. Few of them, however, have also considered the role of the temporal distance of the simulated events. D’Argembeau et al.^19^ looked at temporal distance effects on EFT, and reported differential neural activations when asking participants to think about positive and negative future events that were chosen the day before, taking place in the near future (< 1 month from the present moment) and far future (> 1 year from the present moment). They showed a greater activation of the anterior ventromedial prefrontal cortex (vmPFC) and of the lateral temporal cortex and left inferior frontal gyrus for far as compared with near future events, possibly related to the emotional and uncertain characteristics of far future representations and to the activation of semantic information concerning anticipated future life periods and long-term goals. Conversely, the caudate nucleus was more frequently involved when thinking about emotional (and especially positive) situations in the near future, conceivably related to the concrete simulations of action plans to achieve rewards.

D’Argembeau and colleagues’ study^19^ focused on the neural activation associated with the subjective experience of pre-experiencing the events, as participants were asked to project themselves, imagining each specific event in as much detail as possible. This aspect of mental time-travelling is known as the *elaboration* process, and it usually comes after a *construction* phase consisting in the process of evoking or generating the specific event. In another fMRI study focusing on elaboration, Addis and Schacter^18^ showed that MTL regions responded differentially to the temporal distance of simulated events, depending on whether the events concerned the subject’s personal past or future. For past events, the temporal distance modulation concerned the right parahippocampal gyrus, whose activation was greater for near as compared with far events. For future events, however, a greater bilateral hippocampal activation was found for far as compared with near events. The observation that MTL regions from the common neural network of EAM and EFT respond differentially to the temporal distance of past and future events led the authors to suggest that this core network can be distinctly recruited for past or future simulation^18,39^, probably reflecting different levels of hippocampal integration of contextual details.

Among neuroimaging techniques, electroencephalography (EEG) has a far better temporal resolution than fMRI, allowing for more precise observations of the temporal dynamics of the cognitive processes studied. In a recent EEG study exploring disorientation in Alzheimer's disease, Dafni-Merom et al.^40^ showed that one’s orientation to different places, events and people elicited a comparable brain topography as early as around 200 ms (as compared with a control condition), effects that could not have been observed on a fMRI study. Compared to the fMRI literature, however, studies using EEG to analyze the brain activation patterns of mental time travel to the past and the future are scarce^41–44^. In one of these studies, Weiler and colleagues^41^ compared the slow cortical potentials of the elaboration of past and future events. In early steps of the elaboration process, the authors observed differences at temporo-parietal and parieto-occipital sites, probably reflecting differential recruitment of sensory and semantic detail elaboration for past and future events. During late elaboration, differences concerned frontal sites, presumably signaling differences in monitoring demands.

To the best of our knowledge, however, the EEG literature exploring the neural correlates of mental time travel to past and future situations is not only rare, but it has also overlooked the role of temporal distance. The study by Lavallee and Persinger^45^, in which standardized low resolution electromagnetic tomography (sLORETA) was used to identify electrophysiological correlates of (p)re-experience affect-laden and neutral events, could be considered as an exception. In this study, neutral events showed a different pattern of activation as a function of their temporal distance from the present that changed depending on whether the events were in the past (for which a greater delta power was associated with near events, whereas far events were associated with greater theta power) or the future (for which a greater gamma power was associated with near as compared with far events). Nevertheless, participants from this study imagined future events closer to the present moment as compared with past events, which could hinder the comparison and interpretation of the results.

Importantly, EEG studies in the domain of EAM and EFT can take advantage of the large body of evidence concerning the event-related potentials (ERP) of episodic memory. ERP provide a continuous measure of processing anchored to the event of interest (whether it is a sensory stimulus or a psychological process), making it possible to determine which stages of processing are impacted by the experimental manipulation, as well as enabling the study of cognitive processes that do not elicit a behavioral response^46^. In ERP studies of episodic memory, a classical distinction is made between the subjective experience and neural correlates of retrieving a previously studied item or experienced event, and retrieving the contextual information attached to this episode as well (familiarity versus recollection, see^47^ for a review). In particular, the retrieval of contextual information associated to the event is associated with the *late positive component (LPC)*, also called the parietal ‘old/new effect’, a left parietal component observed between 500 and 800 ms after stimulus onset that is greater for items correctly identified as previously studied than for new items, and that can also reflect the strength of the memory trace or the depth of encoding^48–53^. Some previous ERP studies have observed that this component can also be found when thinking about the future^54,55^; therefore, it is plausible that it reflects the episodic processes underlying events’ simulation regardless of their temporal orientation^1,39,56^.

The ERP literature on episodic memory has also reported a late positive right-frontal component emerging at around 600 ms and lasting up to 2000 ms post-stimulus—the *late frontal effect (LFE)*—that is believed to reflect the ongoing evaluation and monitoring of the retrieved outcome^48,57–61^. The LFE is thought to reflect further monitoring processing when recollection is difficult, poor, or needs additional information to be achieved^62,63^. Importantly, we propose that mental time travel to both the past and future directions may be subject to this monitoring process; as once the event is simulated, its content and qualitative characteristics (i.e., spatiotemporal and perceptual details, affective information, etc.) need to be evaluated in light of the behavioral goals and task demands.

In the present study, we analyze for the first time the ERP associated with the simulation of personal past and future events (i.e., LPC and LFE) as a function of temporal distance. Using the Galton-Crovitz cueing technique^64^, a long-standing method for prompting autobiographical events starting from a word cue, we asked a group of healthy young participants to evoke past and future events at two temporal distances from the present moment: between 1 week and 1 month away (i.e., near), and more than 5 years away (i.e., far). At the behavioral level, we expected to replicate previous findings showing that past events are more detailed than future events. In parallel, a decrease in episodic details as temporal distance of the events increases was also expected in both temporalities^17^. Correspondingly, we expected the LPC to be larger for past events as compared with future events, as well as for near as compared with far events (regardless of the temporal direction). For the LFE, we expected a greater amplitude for future than for past simulations, due to a wider deployment of post-simulation monitoring processes for EFT contents than for EAM.

## Methods

### Participants

Twenty-one participants volunteered and gave their informed written consent to take part in the study. Data from two participants were excluded as they did not complete the task, and data from three additional participants were excluded because their EEG data presented a bad signal to noise ratio. Therefore, data from 16 participants (ages 19–31 years: mean 24, SD 3.23; 9 females; level of education 14–17 years: 15 ± 1.42) were included in the data analyses.

All participants were right-handed French native speakers and had a normal or corrected-to-normal vision. They reported no history of neurological, cardiovascular, or psychiatric conditions, and they scored less than 55 on the STAI-b (State-Trait Anxiety Inventory) questionnaire^65^ (presenting from very mild to moderate levels of trait-anxiety, scores 25–53, mean 39, SD 9.46), and no difficulty for visual mental imagery capacity on the Paivio Individual Differences Questionnaire^66^ (scores 15–23, mean 19, SD 2.37).

Participants were recruited through announcements at the Psychology Faculty of the University of Paris, and they were offered course credits or monetary compensation in exchange for their participation. The study was approved by the Ethics Committee of the Psychology Faculty of the University of Paris and was carried out in accordance with the Declaration of Helsinki.

### Materials

Stimuli consisted of 66 common words in French (4 lists of 15 words each for the experimental trials, plus 6 practice words) from the Lexique database^67^. Stimuli were previously validated on a pilot study in which 45 participants rated the valence and concreteness of 200 words and reported whether they were able to evoke a personal specific past and future event related with each word. For the present experiment, only the words matched in concreteness and in valence (positive, negative, and neutral), and that demonstrated to evoke specific past and future events were selected.

### Procedure

**Figure 1:**
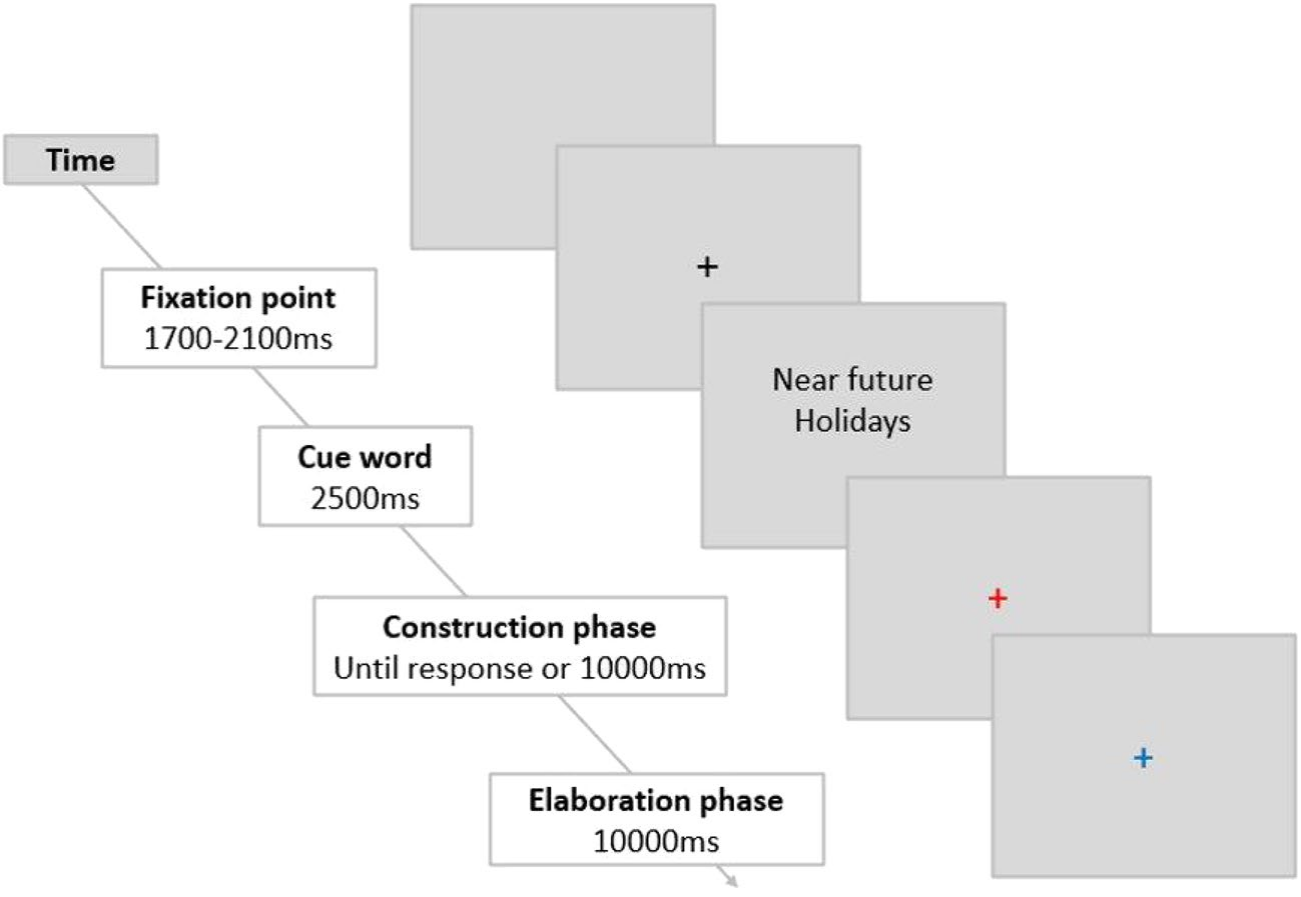
Stimuli and timing in a given experimental trial. Participants had up to 10 s to evoke a unique event related to the word cue (construction phase). They were asked to terminate the construction phase by pressing the spacebar as soon as they had generated the event, which led them to the elaboration phase (10 s long), in which they had to fill the event with as much detail as possible.

The experimental task was run on E-Prime 2.0^68^. Participants observed the screen from a distance of ∼ 57 cm on a 19-inch display. Courier New letter type, 26 letter size was used. At the beginning of each trial, a fixation cross was presented for a variable period of either 1700 or 2100 ms (see Fig. 1). Next, the French words for the experimental condition (far past, near past, far future, or near future) and the word stimuli (for instance: holidays, flower, hospital) were presented above fixation for 2500 ms. Stimuli were replaced by a red fixation cross signalling the construction phase, in which participants had to try to evoke a specific (unique and with distinct spatiotemporal characteristics) event and press the spacebar when they had succeeded in doing so. The construction phase ended when participants pressed the spacebar or after 10 s if no response was given. Then, a blue fixation cross marked the start of the elaboration phase, lasting 10 s, in which participants were asked to mentally evoke the chosen event with as many details as possible.

Participants performed one out of four versions of the task. In each version, a word list was assigned to two temporal conditions and presentation orders across two experimental blocks. That is, each word was presented twice during the experiment; however, we controlled that cue words were not presented twice in the same temporal direction (i.e., past or future) or in the same temporal distance (i.e., far or near). The experimental conditions that could share cue words were therefore: far past and near future; and near past and far future (the order of list presentation was randomized within the experimental blocks). Participants were informed that each word would be presented twice—each time associated with a different time distance and temporal condition—and were asked to evoke different events for each word presentation. Participants were asked to simulate events that were personal, specific in time and place, unique (i.e., not a routine or recurring event), lasted no longer than one day, and presented spatiotemporal, factual (actions, people involved, circumstances), and phenomenal (perceptions, thoughts, emotions) details. Although participants performed the experimental trials silently, during practice trials they were asked to describe out loud the simulated events to check that they met the above-mentioned criteria. On these trials, feedback was provided on the episodic nature of each evocation in order to inform the participants if their simulations needed to be more specific and/or detailed. As a control, after each experimental block participants were asked to rate four events of each temporal condition (randomly chosen by the experimenter among the trials in which participants had succeeded in constructing an event) on several episodic characteristics, such as the uniqueness and duration of the events, their level of spatiotemporal and phenomenological detail, their emotional valence and personal significance, and the intensity of the subjective experience (TEMPau scales^9,27^, see Table 1). These measures were taken only in a sample of eight trials per condition (four by condition and experimental block), in order to have a rapid check of the level of specificity and phenomenology of the simulated events. To this regard, and based on the mentioned control check, participants seemed to respect the task instructions. The date at which these events had taken place or should take place was also noted (in years for far past and future events, and in months, weeks or days for near past and future events). For the sake of simplicity, the reported temporal distance of near events was then converted to a unique measure of days from the present moment.

**Table 1:** **TEMPau scales**. Participants indicated the phenomenological characteristics of eight events from each temporal condition.

Questionnaire items		Scale
Spatial details	Was the event placed in space (place, address, egocentric position in the place)?	0–10 Not at all—Very precisely
Temporal details	Was the event placed in time (date or age, season or time)?	0–10 Not at all—Very precisely
Phenomenal details	Were there any phenomenological/internal details (thoughts, emotions, perceptions)?	0–10 Not at all—Very precisely
Emotional valence	Was evoking the event positive, negative, or rather neutral?	0–10 Really negative—Really positive
Subjective experiencing	Could you almost re-experience/pre-experience the event as if it was here and now, or was it rather vague?	0–10 Very vague—Very detailed
Personal significance	Was this or will this be a significant event in your life (important for your identity or personality)?	0–10 Meaningless—Very important
Date of the event	When did or will the event take place?	–

### Electroencephalography data acquisition and pre-processing

EEG was recorded with a 64-channel active electrode system (Brain Products GmbH) embedded in a nylon cap (10–10 system). Two pairs of electrodes mounted at the outer canthi of both eyes and above and below the left eye were employed to monitor vertical and horizontal eye movements. The continuous EEG signal was acquired at a 500 Hz sampling rate using an Fz reference. The impedance was kept below 50 kΩ. The data were low-pass filtered online at 100 Hz.

EEG data preprocessing and analyses were conducted using EEGLAB^69^ and Fieldtrip^70^ toolboxes and in-house Matlab code (Matlab version 2019b, https://www.mathworks.com/). Data were downsampled at 250 Hz, and offline filtered with a high pass cutoff at 0.1 Hz. Cleanline plug-in from EEGLAB was used to reduce electrical noise at 50 Hz. ICA was carried out on a subset of data high-pass filtered at 1 Hz to ensure a better identification of noisy components; then this decomposition was applied to the 0.1 Hz filtered data (see also^71,72^). Components representing eye movements and blinks were eliminated from the data, and data were then low-pass filtered at 35 Hz. Data from the construction phase were segmented into epochs of 6 s (from − 1000 ms prior to, to 5000 ms after the presentation of the word cue). Only trials in which participants successfully retrieved or imagined an event during the construction phase (and therefore pressed the spacebar) were considered for EEG analyses. Badchannel spherical interpolation was performed on the segmented data to reduce contamination from noisy channels (from 0 to 2 interpolated channels per subject, M = 0.94, SD = 0.57). Finally, data were re-referenced to the mean average. All data segments were baselined to the mean amplitude over 500 ms prior to stimulus onset. Extremely large potential fluctuations, improbable trials and trials in which relative kurtosis was larger than 6 SD were rejected through EEGLAB’s automatic epoch rejection function^63^. In addition, data segments were visually inspected to remove other artifacts, resulting in the following segments included by experimental condition (M ± SD): far past 25.56 ± 1.71, near past 23.69 ± 2.82, far future 25.63 ± 1.96, near future 24.13 ± 3.98.

Electrodes of interest for the LPC and LFE were selected according to previous literature and visual inspection. For the LPC, a subset of three posterior parietal electrodes was chosen (P3/4 and Pz), where this component is usually observed^55,63,73–75^. The LFE, on the contrary, is usually reported on frontal electrode sites^52,63,76^, and therefore a subset of three frontal electrodes (F3/4 and Fz) was selected for its analysis.

### Statistical analyses

A repeated measures ANOVA was conducted to test the effect of time (past and future) and distance (far and near) on participants’ capacity to construct an event –number, as well as reaction time (RT) of successfully retrieved or imagined events– based on the spacebar responses. Finally, for the small subset of simulated events for which the TEMPau scales were filled in, the events’ level of spatiotemporal and phenomenal detail, their valence and importance to the self, and the intensity of the subjective experience were analyzed through a series of repeated measures ANOVA. Significant main effects or interactions were further analyzed through Bonferroni post-hoc analyses. As a control check, separate independent sample t-tests were carried out to verify that the temporal distance of the events in the near and far conditions (in days for near conditions, and in years for far conditions) was not different across each temporal direction (past or future).

In the present study, only the construction phase was analyzed, as the ERP of interest have only been reported at this stage of processing. Separate repeated measures ANOVAs were performed on the two components of interest, related to the episodic construction of the events: the LPC and the LFE. For the LPC, a 2 × 2 × 3 ANOVA was conducted, with time (past and future), distance (far and near) and electrode (P3, P4, and Pz) as factors. For the LPC analysis, mean amplitudes from 500 to 800 ms across the selected parietal electrodes were taken, similarly to previous studies^55^. The 2 × 2 × 3 ANOVA for the LFE included time (past and future), distance (far and near) and electrode (F3, F4, and Fz) as factors. The temporal window in which the LFE mean amplitudes were analysed comprised from 800 to 2000 ms. Greenhouse–Geisser sphericity correction was used for comparisons including factors with more than two levels (i.e., the electrode factor). Significant interactions were further analyzed through Bonferroni post-hoc analyses.

In order to be able to determine the existence or absence of an effect over the temporal windows of the ERP of interest, non-parametric cluster-based permutation analyses were performed^77^. For these analyses, and following the main aim of the present study, only the temporal distance effect (separately for the past and future temporalities) was assessed. First, data entering the cluster-based analyses were reduced to the time dimension, as amplitude from each time sample from the LPC and LFE time windows (500-800 ms for the LPC, 800-2000 ms for the LFE) was averaged across the electrodes defined a priori for each component (P3/4 and Pz for the LPC analyses, F3/4 and Fz for the LFE analyses). Cluster-based permutation t-tests were performed for the comparison between far and near events in the past and in the future dimension (i.e., far past vs near past; and far future vs near future comparisons) for each component of interest. For all contrasts, a t-test was performed for each time sample. Adjacent time-points with p-values below 0.05 were grouped into clusters. For each cluster, cluster-based statistics were computed as the sum of their t-values. In order to establish the significance of the cluster statistic, Monte Carlo estimates were calculated by means of permutation tests (n = 1000) under the permutation distribution of the maximum cluster-level statistic. P-values for each cluster were determined as the proportion of permutations above the observed cluster-based statistic.

## Results

### Behavioral results

The repeated measures ANOVA for the total number of trials in which participants succeeded in constructing an event showed a main effect of distance, *F*(1,15) = 12.794, *p* = 0.003, *η*_*p*_^*2*^ = 0.460, as there was a greater number of constructed events for far as compared with near conditions (see Table 2 for the mean and SD of the behavioral variables analyzed). No other main effects or interactions reached statistical significance (all *p*s > 0.413). The analysis of the RT during event construction (for trials in which an event was successfully constructed) did not reveal any statistically significant main effects or interactions (all *p*s > 0.113), indicating that participants took the same amount of time to retrieve or imagine the events regardless of the temporal condition (past and future) and of the temporal distance (far and near).

**Table 2:** **Behavioral data**. Mean (standard deviation in parenthesis) number of simulated events, reaction time, and responses to the TEMPau scales as a function of time and temporal distance.

	Far past	Near past	Far future	Near future
Number of simulated events	28.81 (2.04)	25.63 (5.16)	28.63 (1.86)	26.50 (4.31)
Reaction time (ms)	3377.14 (1222.27)	3877.11 (1206.18)	3558.48 (1404.60)	3681.30 (1271.12)
Level of spatial detail	8.51 (1.12)	9.12 (1.00)	6.49 (1.54)	7.34 (1.43)
Level of temporal detail	7.03 (1.17)	8.39 (1.08)	4.45 (1.61)	5.88 (1.21)
Level of phenomenal detail	7.24 (1.76)	7.15 (1.39)	7.14 (1.60)	6.18 (1.75)
Emotional valence	5.86 (0.92)	6.53 (1.14)	6.71 (0.99)	6.58 (1.09)
Subjective experiencing	7.11 (1.28)	7.80 (1.29)	6.31 (1.42)	6.27 (1.60)
Self-relevance	6.19 (1.78)	5.34 (1.85)	6.89 (1.70)	5.32 (1.87)
Distance from the present (years)	9.75 (3.21)	–	12.46 (7.96)	–
Distance from the present (days)	–	54.82 (52.50)	–	41.58 (30.80)

The repeated measures ANOVA carried out on the control subset of constructed events for the TEMPau scales showed that *spatiotemporal details*, for example, were separately modulated by time (whatever the distance) and distance (whatever the time). The level of spatiotemporal detail was greater for past events compared to future events (*F*(1,15) = 66.184, *p* < 0.001, *η*_*p*_^*2*^ = 0.815 for spatial, and *F*(1,15) = 71.287, *p* < 0.001, *η*_*p*_^*2*^ = 0.826 for temporal details), and for near as compared with far events (*F*(1,15) = 9.625, *p* = 0.007, *η*_*p*_^*2*^ = 0.391 for spatial, and *F*(1,15) = 27.333, *p* < 0.001, *η*_*p*_^*2*^ = 0.645 for temporal details). The interaction effect was not significant (both *p*s > 0.535).

A slightly different pattern of results was observed for *phenomenal details* (internal thoughts, emotions or perceptions), for which again separate main effects of time (*F*(1,15) = 6.157, *p* = 0.025, *η*_*p*_^*2*^ = 0.291) and of distance (*F*(1,15) = 14.145, *p* = 0.002, *η*_*p*_^*2*^ = 0.485) were observed. As for the level of spatiotemporal details, participants reported a greater level of phenomenal details in the past as opposed to future events (regardless of the events’ distance). However, the level of phenomenal details was greater for far as compared with near events (regardless of the simulation direction). The interaction effect did not reach statistical significance (*p* = 0.063).

The manipulation of the time and distance of the events did not modulate their *emotional valence* (all *p*s > 0.060). In contrast, the *personal significance* of the events showed a significant main effect of distance (*F*(1,15) = 28.632, *p* < 0.001, *η*_*p*_^*2*^ = 0.656), as far events were rated as more important for the self than near events, regardless of the simulation direction. No other main effects or interactions reached statistical significance (all *p*s > 0.166).

For the *subjective experience* of the events’ evocations, a significant main effect of time (*F*(1,15) = 23.013, *p* < 0.001, *η*_*p*_^*2*^ = 0.605), and of distance (*F*(1,15) = 9.329, *p* = 0.008, *η*_*p*_^*2*^ = 0.383) were found, revealing a higher intensity of mental (p)re-experiencing for past as compared with future events, as well as for near as compared with far events. However, the interaction between time and distance factors was also significant, *F*(1,15) = 5.325, *p* = 0.036, *η*_*p*_^*2*^ = 0.262. Post-hoc analyses showed that near past events were rated as being more vividly re-experienced as compared with far past events (*p* = 0.045). However, this comparison was not significant for near future and far future events (*p* = 1). Near past and far past events also presented a greater sense of subjective experiencing than both near future and far future events (all *p*s < 0.018).

Finally, none of the independent sample t-tests concerning the *temporal distance* of the events as a function of temporal direction reached statistical significance (all *p*s > 0.236), indicating that the past and future events evoked were placed at similar temporal distances from the present moment (M ± SD: 11.29 ± 5.10 years for far events; and 48.20 ± 33.57 days for near events).

### Electrophysiological results

**Figure 2:**
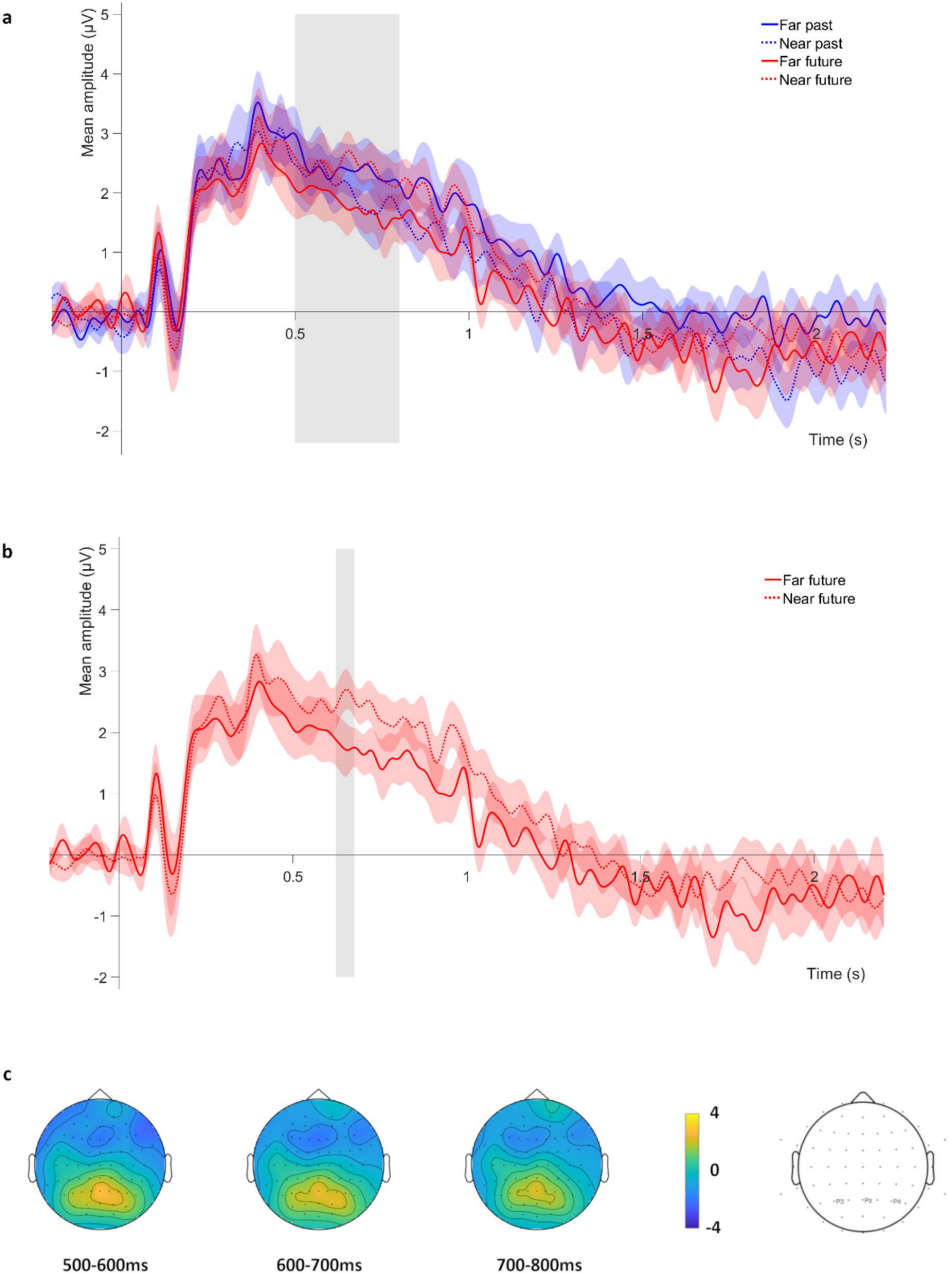
Electrophysiological data for the LPC time window. (**a**) Mean amplitude of the ERP averaged across the three electrodes selected for the LPC analyses (P3/4 and Pz). The grey rectangle shows the time window of this component (from 500 to 800 ms). (**b**) Far future vs near future comparison for the LPC, grey rectangle shows the cluster of time points for which the comparison was significant. For (**a**) and (**b**), shadowed regions indicate the standard error of the mean for each experimental condition. (**c**) Scalp map of the LPC for the mean average of all experimental conditions, showing the temporal course of this component.

The ANOVA for the time window of the *LPC* revealed a significant time × distance interaction, *F*(1,15) = 6.280, *p* = 0.024, *η*_*p*_^*2*^ = 0.295 (see Fig. 2a). However, none of the post-hoc comparisons survived the Bonferroni correction (all *p*s > 0.206). A significant distance × electrode interaction was observed for the LPC time window, *F*(1.41,21.17) = 4.629, *p* = 0.032, *η*_*p*_^*2*^ = 0.236. However, post-hoc comparisons did not survive Bonferroni correction (all *p*s = 0.260). No other main effects or interactions reached statistical significance (all *p*s > 0.235).

**Figure 3:**
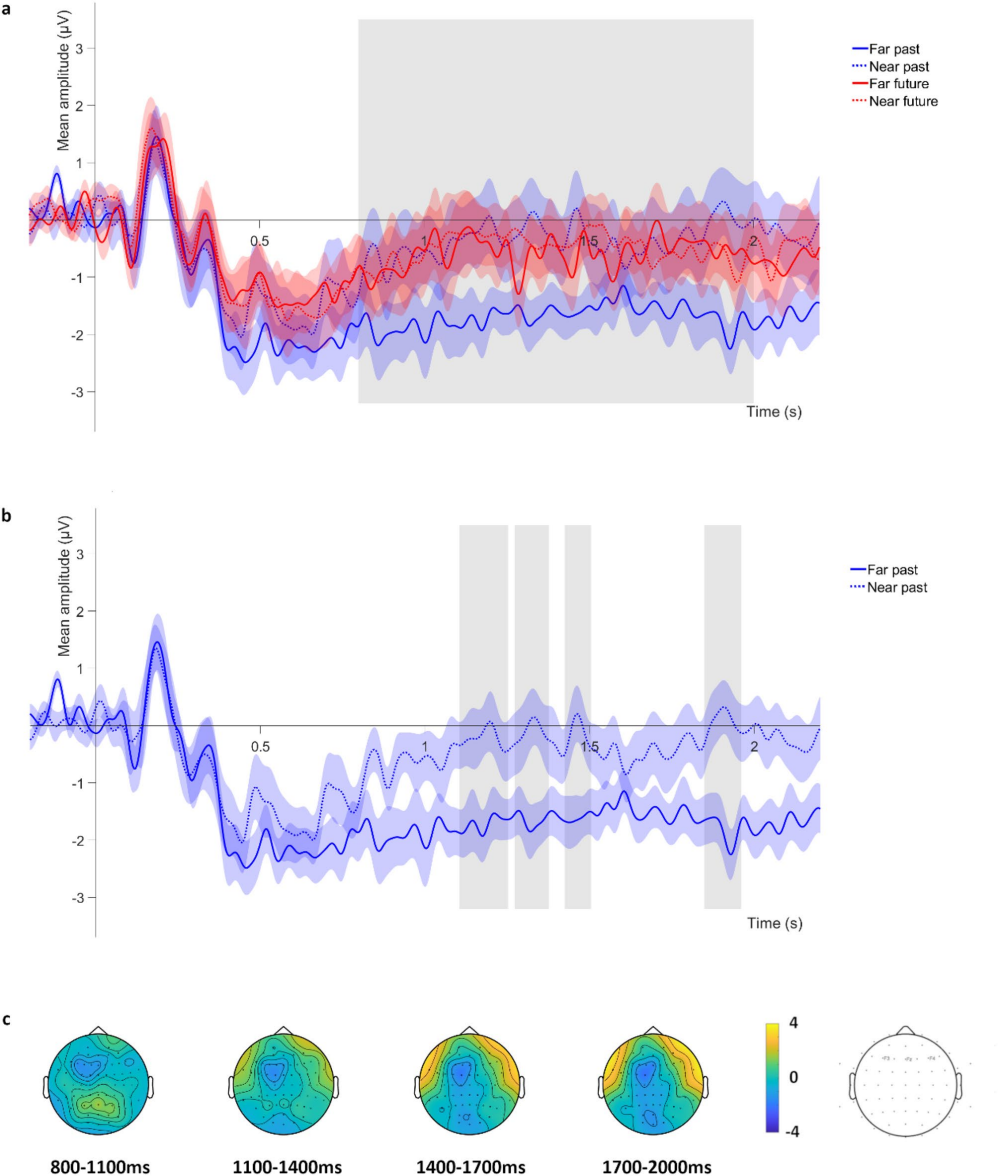
Electrophysiological data for the LFE time window. (**a**) Mean amplitude of the ERP averaged across the three electrodes selected for the LFE analyses (F3/4 and Fz). The grey rectangle indicates the time window of this component (from 800 to 2000 ms). (**b**) Far past vs near past comparison for the LFE, grey rectangles show the clusters of time points for which the comparison was significant. For (**a**) and (**b**), shadowed regions indicate the standard error of the mean for each experimental condition. Panel (**c**) displays the temporal course of the LFE, averaged across conditions.

For the time window of the *LFE*, the ANOVA showed a main effect of distance, *F*(1,15) = 5.827, *p* = 0.029, *η*_*p*_^*2*^ = 0.280, as mean amplitudes for this component were greater in near as compared with far events (see Fig. 3a). The electrode main effect reached statistical significance, *F*(1.61,24.19) = 5.430, *p* = 0.016, *η*_*p*_^*2*^ = 0.266. Post-hoc Bonferroni analyses revealed greater amplitudes for the F4 electrode as compared with both the F3 and Fz electrodes (both *p*s < 0.037). A time × distance interaction was also observed, *F*(1,15) = 5.986, *p* = 0.027, *η*_*p*_^*2*^ = 0.285 (Fig. 3a). Post-hoc comparisons revealed that the LFE mean amplitudes in the far future, near future and near past were larger than in the far past condition (all *p*s < 0.046). No other main effects or interactions reached statistical significance (all *p*s = 1). A representation of the temporal course of the two components of interest across the electrode scalp can be seen in Figs. 2c and 3c.

Cluster-based analyses for the LPC time window revealed no significant effects for the far past vs near past comparison (no clusters were formed). By contrast, for the far future vs near future comparison a significant effect was found, which corresponded to a cluster extending from 624 to 676 ms after stimulus presentation. For this cluster, the near future condition elicited a larger LPC component than the far future condition (*p* = 0.034, see Fig. 2b).

For the LFE, the cluster-based analysis comparing near past and far past events revealed significant effects extending over the temporal windows of 1104–1252 ms, 1272–1376 ms, 1424–1504 ms, and 1848–1960 ms. For the four significant clusters found in this comparison, near past events elicited a larger LFE component than far past events (all *p*s < 0.038, see Fig. 3b). Lastly, for future events, the near vs far comparison was non-significant (*p* value of the only formed cluster = 0.909).

## Discussion

The present study explored for the first time the electrophysiological correlates of the construction of personal past and future events as a function of their temporal distance from the present moment. Following the claim that episodic simulation enabling both EAM and EFT could rely on the same constructive processes^1^, and in line with previous studies on semantic and episodic contributions to past, present and future self-knowledge^54,55^, we extended the LPC—a well-known episodic memory component—to future event simulation. In a similar manner, and for the first time, to the best of our knowledge, we extended the LFE to future simulation, arguing that the monitoring processes reflected by this component—oriented to evaluate the event’s content and qualitative characteristics—would not be restricted to the simulation of past events but could also be observed in future simulation.

First, according to our check of a subset of evocations, participants seemed to have adhered to task instructions regarding the temporal distance of the events, as simulated events in the past and future did not differ in their distance from the present moment (contrary to the study by Lavallee and Persinger^45^), therefore allowing for the comparison of past and future simulation. Based on all the evocations, we observed that participants succeeded better in simulating events in far (more than 5 years away from the present moment) as compared with near (between one week and one month away from the present moment) temporalities. This difficulty in constructing unique and specific events in near as compared with far situations could be due to the difference in width of the two periods (the near condition period being more constrained than the far one). Finally, the reaction time required for event construction was not modulated by time nor distance, suggesting that participants succeeded in simulating an event if they completed the simulation around this temporal window (around 3500 ms from the presentation of the word cue), and that trying to do so beyond this time period would result in a failure to simulate an event (which happened more often for near temporalities).

Concerning the nature of the simulated events for which the TEMPau scales were filled in, coherent with a prior body of research^9,17^, past events from our study presented a greater level of spatiotemporal and phenomenological details than future events, and were associated with a greater intensity of mental experience. As expected, a greater level of spatiotemporal detail and intensity of (p)re-experiencing was also found for near as compared with far events. Contrary to our expectations, however, we found that the level of phenomenological details was greater for far as compared with near conditions (irrespective of the time condition). A plausible explanation for this unexpected result is that, as far events were rated as more personally relevant than near events, participants’ phenomenological ratings could have been shaped by the events’ personal importance. On the other hand, the finding of a greater level of phenomenological details for far events stands in contrast with the greater intensity of (p)re-experiencing found for near as compared with far events. It should be considered that, apart from the number of phenomenal details, other factors such as spatiotemporal and factual details modulate (p)re-experiencing intensity. Therefore, this pattern of results in our study could have been mediated by other factors, such as the level of spatiotemporal detail (which was also greater for near events). Finally, although they are mainly consistent with previous research, data on the nature of the simulated events should be considered with caution, as they were only collected for a small sample of evocations for control purposes.

The electrophysiological markers of interest were differently modulated by the temporal distance of the events, depending on whether the simulation concerned the past or future domains. Concerning the LPC, a component thought to reflect the amount of information retrieved^78,79^ and that has been largely associated with the episodic nature of recollection^47,73^, an effect of temporal distance was found for future events, as this component was larger for near future as compared with far future events. Future events simulated far away from the present moment seemed to engage in an impoverished episodic simulation processing compared to future events happening near to the present time. This result partially fits the proposal of the TEDIFT model^20^, which predicts a decrease in episodic representations as the events’ temporal distance from the present moment increases, both in the past and the future directions. In this line, a recent single-case study of a semantic dementia patient^80^ showed that he could simulate personal specific events in the near future (i.e., in a week or in a year from the present moment), but that his EFT capacity for the distant future (in 5 years’ time) was severely impaired. Similarly, this patient’s ability to recall near past events (i.e., from a week or a year ago) was preserved, but he presented difficulties in recalling remote (5 years ago) events. Previous fMRI studies have also found differences on far and near future simulation (see for example^18^), although focusing on event elaboration, instead of construction. Even if the temporal resolution limitation of the technique makes us probably observe a later time window of the neural responses to the task, it could be argued that far future vs near future modulations of the LPC also reflect different amounts or kinds of episodic binding, depending on the temporal distance of the events.

Contrary to our expectations, however, the LPC did not show a temporal distance modulation for past events in our study. This pattern of results could be pointing to a more important role of semantic processes in mediating temporal distance effects for the future as compared with past simulation. Indeed, the simulation of future events has been reported to be more abstract (i.e., less rich in contextual and sensorial details) than remembering past events^12,16,17^, as demonstrated also by the subset of evaluated simulated events, whose level of spatiotemporal and phenomenological details and the intensity of (p)re-experiencing was greater for past as compared with future events. Increasing the temporal distance of simulated events in the future could therefore accentuate this abstraction process, which seemed less pronounced for remembering past events at the same temporal distances.

Finally, contrary to our expectations, we did not observe a greater amplitude for past as compared with future events for the LPC. We expected the greater level of episodic detail of past events (with respect to future events) to be reflected by a greater LPC amplitude for past simulation. However, ERP studies in which participants had to decide whether the given personal traits could be applied to their present or future selves have shown comparable LPC amplitudes for past and future self-knowledge and an episodic memory task^55^, as well as greater amplitudes for future self-knowledge as compared with present self-knowledge^54^. Altogether, the described pattern of results seems to indicate that mental time travel to past and future selves or to specific events into the past and future elicit similar LPC activity, which is also comparable to the activity elicited by episodic memory tasks. Further studies should explore the factors explaining this pattern of results.

Concerning the LFE, this component is associated with post-retrieval monitoring processes^59,81^, especially when recollection requires additional effort (i.e., because it is difficult, poor, or needs additional information to be achieved)^62,63^. Coherently with previous studies showing that this component is usually maximal at right frontal electrodes^48,57,59^, in our study we observed greater amplitudes for the LFE at the right-side electrode than at the central and left-side electrodes. Concerning our experimental manipulation, the LFE findings supplement LPC results, as this component was also differently modulated by the temporal distance of the events, depending on the temporal direction of the simulation. In particular, the LFE was found to be larger for near as compared with far events, especially in past event simulation. Moreover, LFE mean amplitudes for near future and far future events were comparable to those of near past events, while far past events did not elicit a comparable LFE. These results suggest that far past event simulation does not depend on post-simulation monitoring processes as much as near past or future simulation. It has been proposed that imagining the future requires more effort than remembering the past, as it entails the flexible recombination of information into a novel, coherent, and plausible scenario, together with the inhibition of inappropriate information^82^. In this vein, the LFE in our experiment was significantly smaller for far past events than for the rest of the experimental conditions, indicating that the construction of past events in far temporal distances did not require an effortful retrieval or subsequent attempts. It is plausible that near past events elicited a larger LFE than far past events because the situations retrieved in near temporal distances were more similar to one another, with far past events being more unique or special. This is consistent with previous literature associating this component with the evaluation and monitoring of the outcome of the retrieval attempt, searching for specific features of details^60,83^. In addition, participants from our study rated the personal significance for the subset of checked events as being greater for far as compared with near events. It is therefore conceivable that far past events presented more self-relevant distinctive details than near past events, or that the contextual details retrieved for past events were better bonded, alleviating the need for ongoing monitoring processes^26,27,31,84^. Finally, the fact that near and far future events elicited a similar LFE seems to indicate that, contrary to past event simulation, the monitoring processes engaged in future event simulation do not depend on their temporal distance from the present moment. Although previous studies have proposed that near and far future simulation differ in terms of action planning or in the events’ levels of uncertainty^19^, it is plausible that the monitoring processes underlying these operations led to similar LFE activations.

It is worth mentioning that the temporal distance comparison in the future condition for the LPC was not statistically significant when the amplitude of the whole time window of interest was averaged, but rather when time points from this period of interest were separately considered by means of cluster-based analyses. Cluster-based permutation analyses are known to maximize the sensitivity to the expected effect by incorporating biophysically motivated constraints in the test statistic (i.e., temporal autocorrelation), while correcting for multiple comparisons^77^. Therefore, even if temporal distance differences on the LFE seem to extend over longer periods and to be more statistically robust (as they survive very strict multiple comparison corrections, such as Bonferroni), cluster-based permutation analyses confidently support the existence of a temporal distance effect for the future simulation over the LPC time window. Regarding the significant clusters that arose from LFE analyses, the present work does not allow to determine whether early (from 1100 to 1500 ms) and late (around 1900 ms) effects correspond to the same or different cognitive processes. In fact, the functions associated with the LFE are subject of debate, and it has been proposed that instead of post-retrieval monitoring processes, right prefrontal activity related to this component would depend on the number of internal decisions that were required prior to a behavioral response^85^. Moreover, some authors have proposed that the generators of the LFE extend beyond response selection, indicating that ‘post-selection’ processes would also be reflected by this component^57^. Although in the present experiment responses were always given after the time window of the analyzed ERP, further research should try to disengage the cognitive and neural generators of the LFE.

In summary, findings from this study highlight the role of temporal distance in mental time travel, supporting theoretical accounts claiming that remembering and imagination are part of the same constructive process^1^, on which the temporal distance of the events can have both common and distinct effects. Evidence from the present work points to a different modulation of episodic and monitoring processes as a function of temporal distance for the past and future directions. On the one hand, episodic simulation was greater for near future than for far future events. On the other hand, post-monitoring processes were more prominent for the simulation of near past events, as compared with far past event simulation, as well as for near and far future as compared with far past simulations. Although in the present study we focused on the episodic component of mental time travel, semantic memory appears to play a pivotal role in past and future simulation. The extent to which temporal distance modulates semantic memory contributions to mental time travel remains an open question. Future ERP studies could address this question by analyzing both more episodic components such as the LPC—which has been shown to be also modulated by semantic processing, especially when it concerns personal knowledge^49,50,68^—and components related to semantic processing, such as the N400^49,51^. For this purpose, events happening at farther temporal distances from the present moment could be targeted, in order to guarantee that some kind of abstraction process has taken place.

## Data Availability

Behavioral data can be downloaded from the Open Source Framework (https://osf.io/m7qh2/). EEG recordings are available upon request from the corresponding author (itsaso.colas-blanco@u-paris.fr).
